# Supplementary material for: Stretchable self-tuning MRI receive coils based on liquid metal technology (LiquiTune)
Source: Sci Rep. 2021 Aug 10;11:16228. doi: 10.1038/s41598-021-95335-6 (PMC8355233; doi:10.1038/s41598-021-95335-6)
Supplement: Supplementary file 3 — Supplementary Information. [file 41598_2021_95335_MOESM3_ESM.docx]

Stretchable self-tuning MRI receive coils based on liquid metal technology

Supporting Information

**Elizaveta Motovilova^1,2,*^ | Ek Tsoon Tan^2^ | Victor Taracila^3^ | Jana M. Vincent^3^ |Thomas Grafendorfer^3^| James Shin^1^ | Hollis G. Potter^2^ | Fraser J. L. Robb^3^ | Darryl B. Sneag^2^ | Simone A. Winkler^1,*^**

^1^Department of Radiology, Weill Cornell Medicine, New York, NY, 10065, USA. ^2^Department of Radiology, Hospital for Special Surgery, New York, NY, 10021, USA. ^3^GE Healthcare, Ohio, USA. * Email: [elm4010@med.cornell.edu](mailto:elm4010@med.cornell.edu); [ssw4001@med.cornell.edu](mailto:ssw4001@med.cornell.edu)

To find optimal coil dimensions, numerical optimization of coil parameters was performed. Figure S1 demonstrates an example of the optimization steps. Figure S1 (a) shows a schematic view of the coil with coil dimensions and parameter names indicated. This coil is then subjected to a 1D stretch along the x-direction. Figure S1 (b) shows the resonance frequency change over a degree of stretch from 0% to 100%, where different colors correspond to different digit lengths *p* (from 2mm to 8mm). The total maximum frequency change Δ*f* is then measured and plotted with respect to the parameter value *p* in Figure S1 (c). It can be inferred from this figure that the frequency change with stretch can be minimized by increasing the digit length *p*. Other parameters can be studied similarly, such as e.g. number of digits *N*, as shown in Figure S1 (d). It can be inferred from this figure that the frequency change with stretch can be minimized by increasing the number of digits *N*. It is even possible to construct parameter maps, where two parameters are changed simultaneously, and the optimal parameter combination can be found. Figure S1 (e) shows a parameter map for a pair of parameters, i.e. trace gap *g* and trace width *w*, where the maximum frequency change with stretch is represented by the color bar. It shows that to minimize resonance frequency change with stretch, both trace gap *g* and trace width *w* have to be minimized.

w


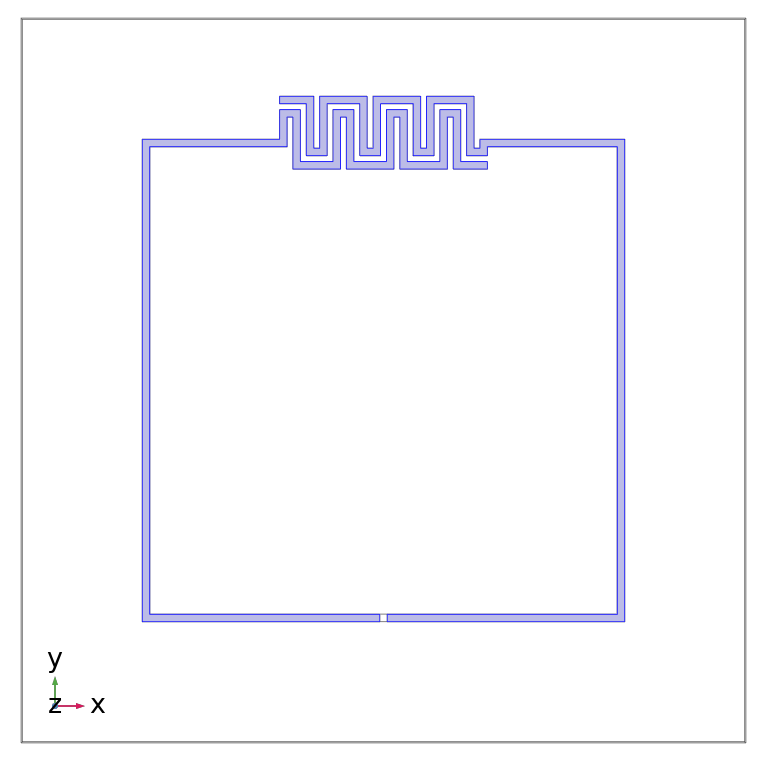


65 mm

65 mm

N

p

Δf

(a)

(b)

(c)

(d)

(e)

Figure S1. Optimization of coil parameters.

These numerical simulations help to better understand the dependencies between different coil parameters (such as digit length *p*, digit number *N*, trace width *w*, trace gap *g*) and desired coil characteristics (such as minimal frequency shift with stretch Δ*f*) and to guide the design process. However, manufacturing capabilities put some constraints on the final design. The digit length p and number of digit *N* cannot be too large as it will increase the coil dimensions. On the other hand, trace width *w* and trace gap *g* cannot be too small as we are limited by the resolution of the 3D printer which fabricates the coil molds. Therefore, a tradeoff has to be found that provides sufficient frequency stability within reasonable coil dimensions. For this proof-of-concept demonstration we found one such design that has the following parameters: number of digits *N=8*, digit length *p=7*, trace gap *g=0.5mm*, trace width *w=0.5mm*. Although this design demonstrates the proposed concept and provides sufficient frequency stability over the specified stretching limit (up to 30%), there are other possible parameter combinations that could provide even better performance. The search for other optimal solutions is beyond the scope of this work.

The RF coil was constructed by filling EGaIn [1] liquid metal in microfluidic channels made of stretchable insulating silicone Ecoflex^TM^[2]. The microfluidic channels were made by bonding two silicone layers, where the top layer contains microfluidic channel features and the bottom layer is featureless. There are several approaches to bond silicones such as oxygen plasma bonding, corona discharge, partial curing, and uncured silicone adhesive technique [3]. For this work we used the partial curing bonding technique which does not require any additional expensive tools required for the oxygen plasma and corona discharge methods.

Figure S2 demonstrates step-by-step instructions for stretchable coil fabrication. The process begins with the fabrication of two 3D printed molds: one containing (a) the negative of the desired coil geometry and the other one (b) being the sealing layer without any features. The mold designs are created in a computer-aided design (CAD) software (SOLIDWORKS®) and the corresponding STEP files are exported to a high precision 3D printer (Prusa i3 MK3S, using a 25µm nozzle and a 50µm layer height) for fabrication using polylactic acid (PLA) material. Next, (c) the Ecoflex^TM^ mixture is prepared by mixing the crosslinker and prepolymer at a ratio of 1
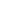
:
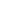
1 (equal parts of A and B of the provided liquid polymer). The two parts are combined in a mixing cup and mixed vigorously for 3 min. The mixture is then (d) placed in a vacuum chamber (BACOENG 3-gallon chamber and pump kit [4]) for degassing until all the air bubbles are removed (approximately 10 min). The liquid polymer is then (e) casted into the mold with the coil features (mold (a)) and left to fully cure. The curing can be performed at room temperature, which will require approximately four hours. However, this process can be sped up by placing the mold with the polymer in an oven or a drying cabinet with temperature control. Here, we used a drying cabinet, which reduces curing time to 25 min at 45°C. After the polymer is fully cured, (f) it can be easily removed from its mold. The fabricated top layer with microfluidic channel features is shown in (g). Next, another batch of Ecoflex^TM^ silicone is prepared by following steps (c)-(d) and casted (h) into the featureless mold (mold (b)). When the silicone in the bottom mold is partially cured (after approximately 8 min at 40°C) the top layer (g) is carefully placed on top of the bottom layer and left to bond and fully cure (approximately 20 min at 45°C). The combined layers are then removed from the mold (j). To create an inlet and an outlet for the microfluidic channels, two medical needles (21 G; inner diameter: 0.5 mm, outer diameter: 0.8 mm) are used to puncture insertion channels through the polymer. Liquid metal (eGaIn) is (k) injected from a 5 mL syringe by positive pressure. Copper wires are inserted into the microfluidic channels and epoxy resin (Sil-Poxy^TM^ by Smooth-On) is used to seal the channel openings. Supporting video V1 demonstrates the flexibility and stretchability of the fabricated coil.


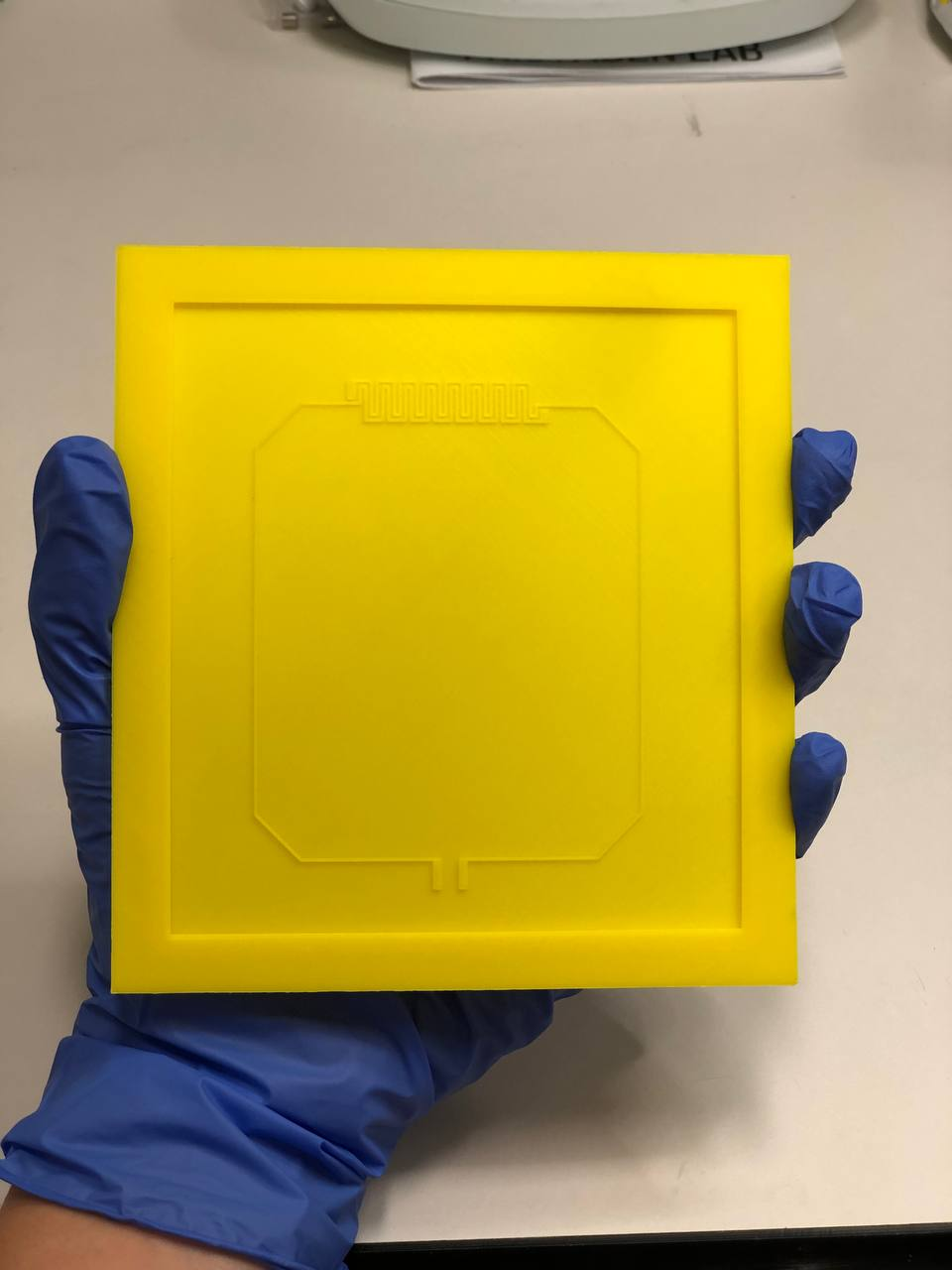

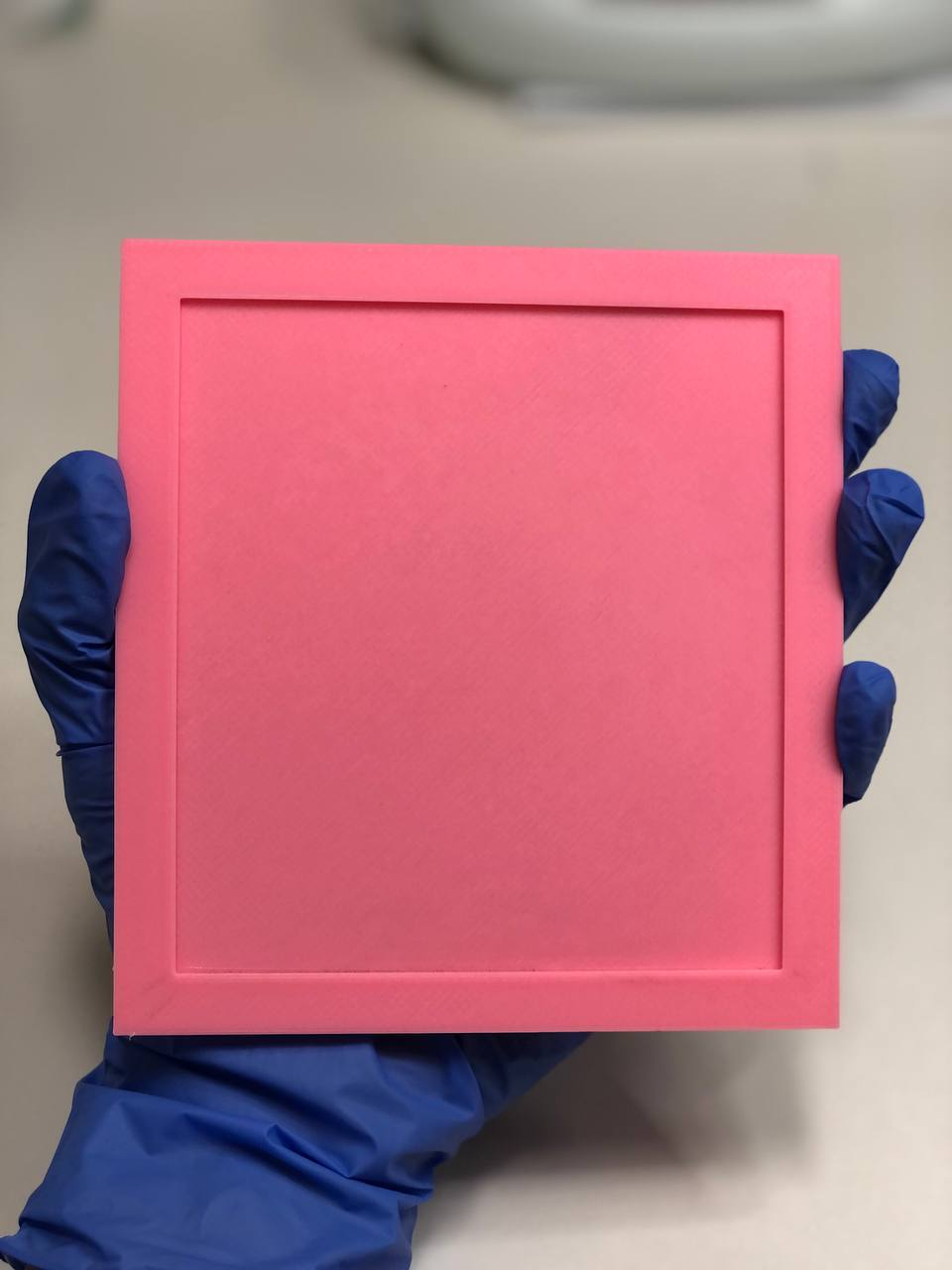

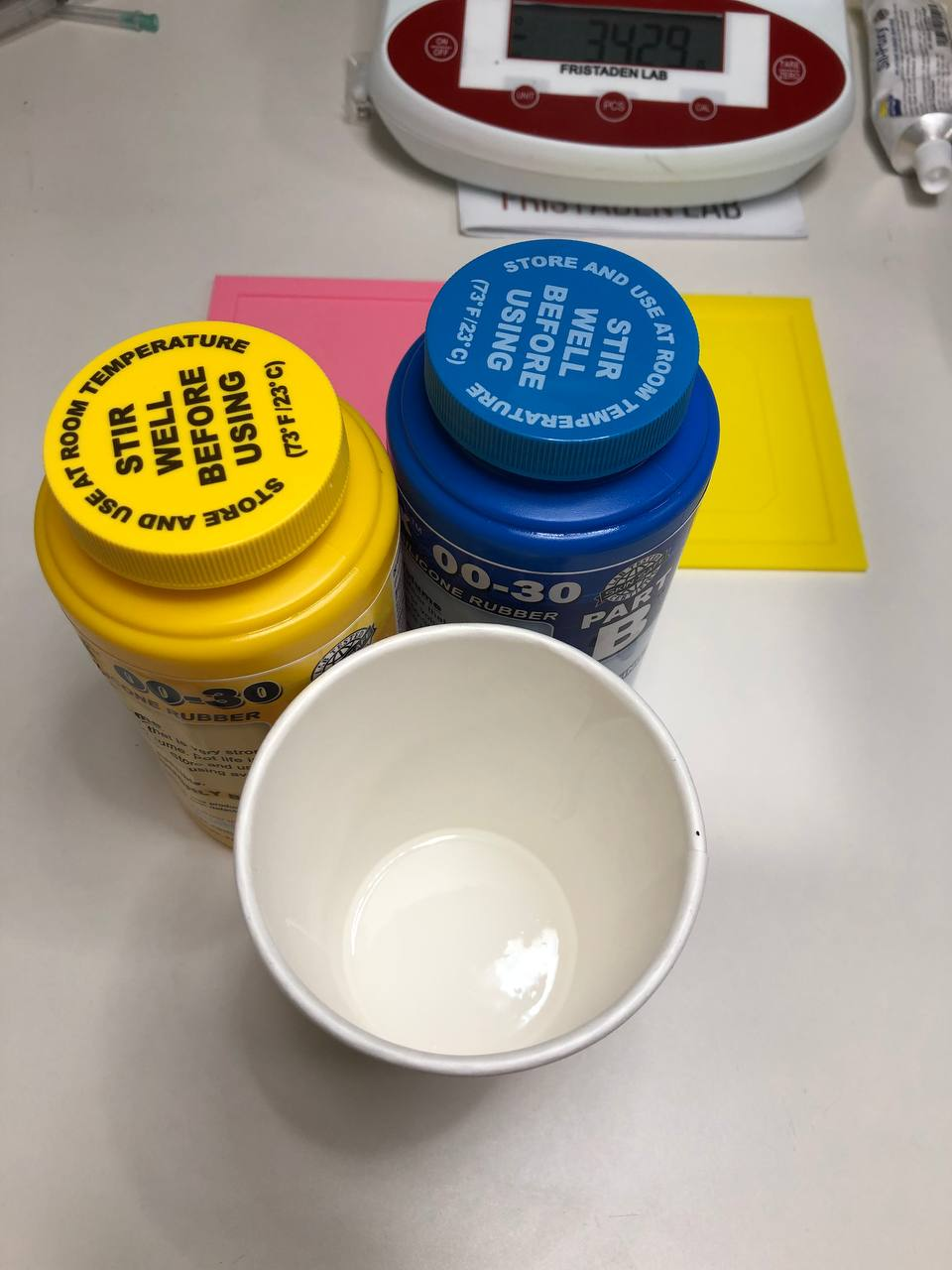

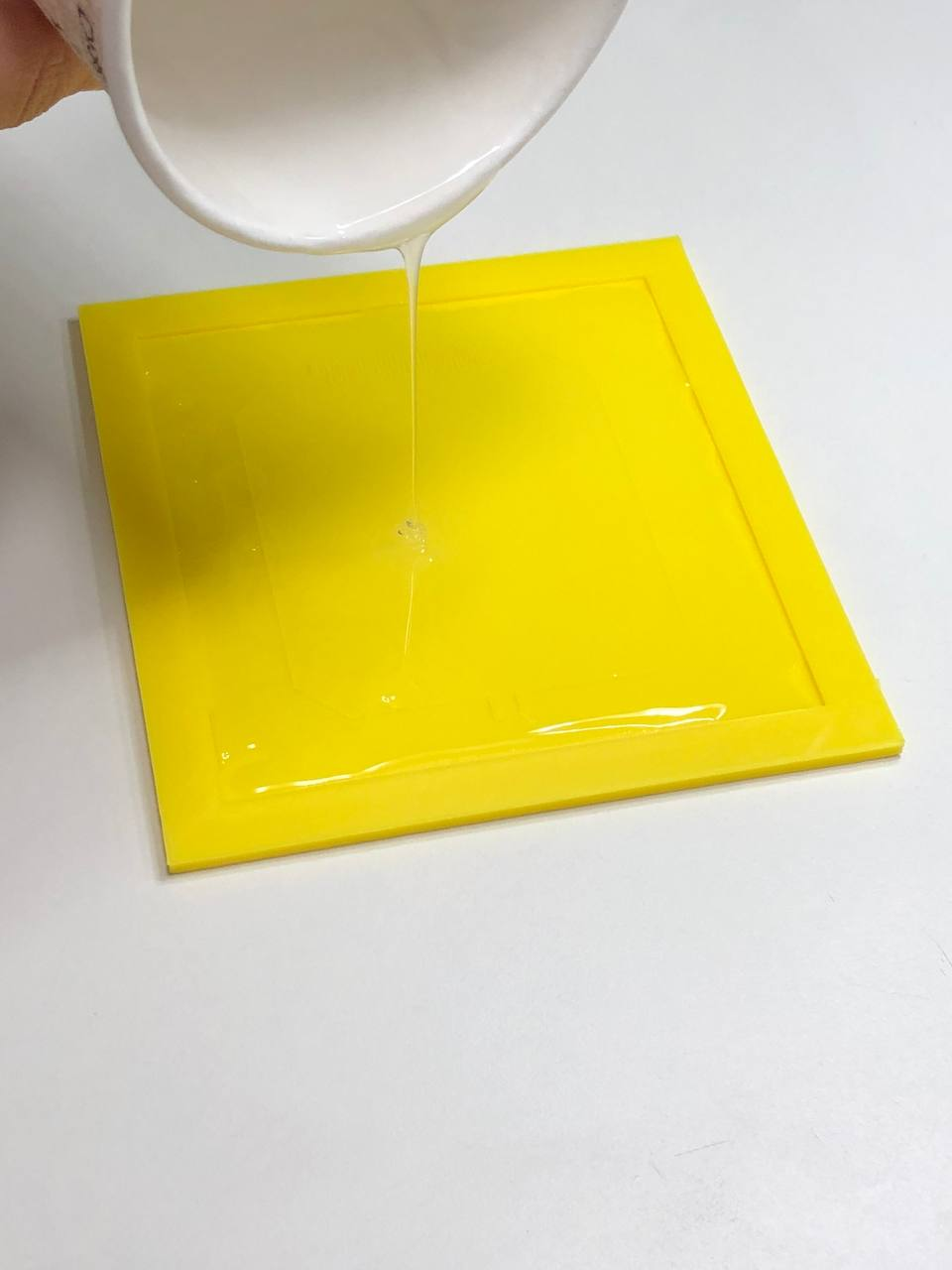

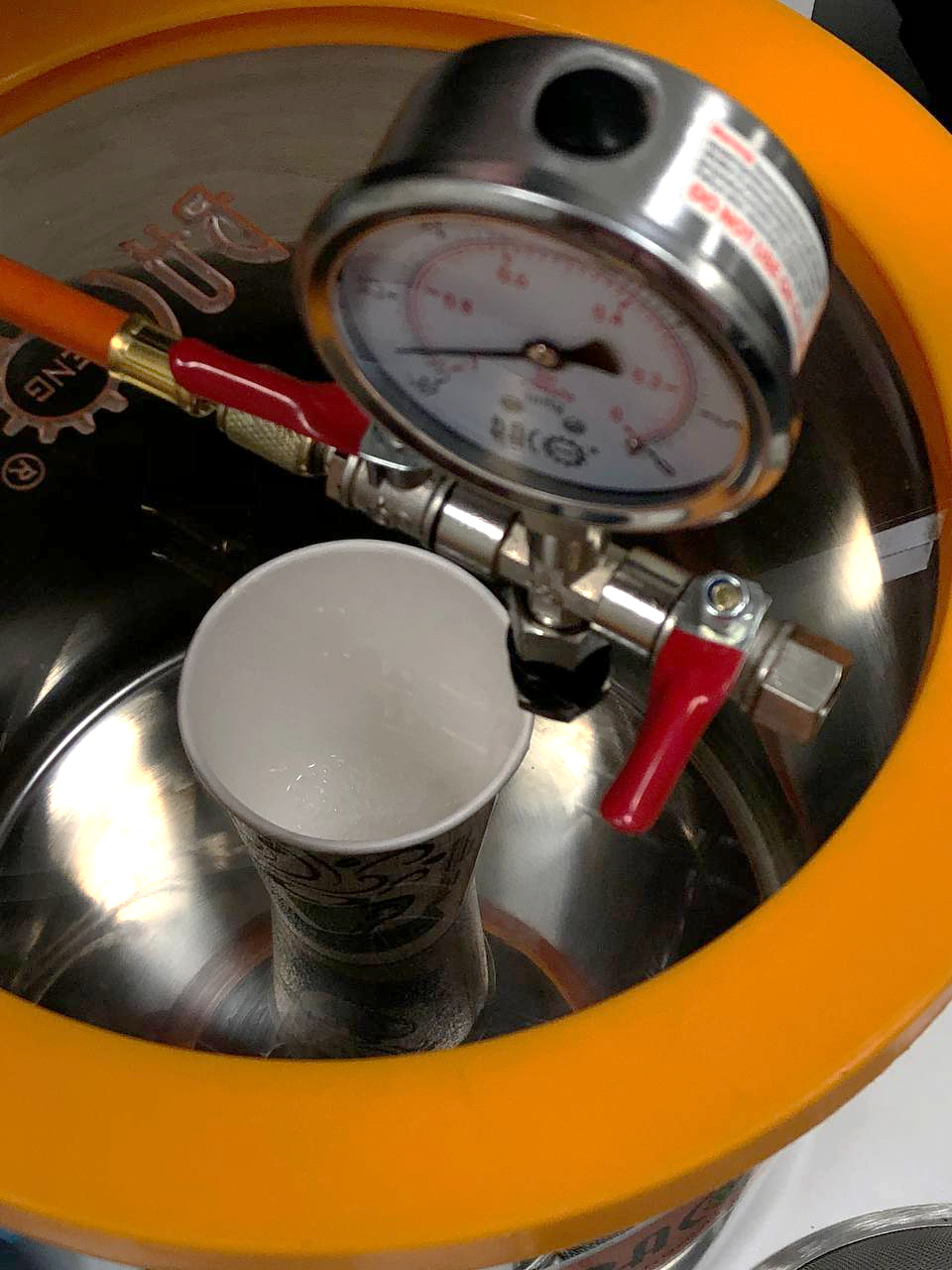

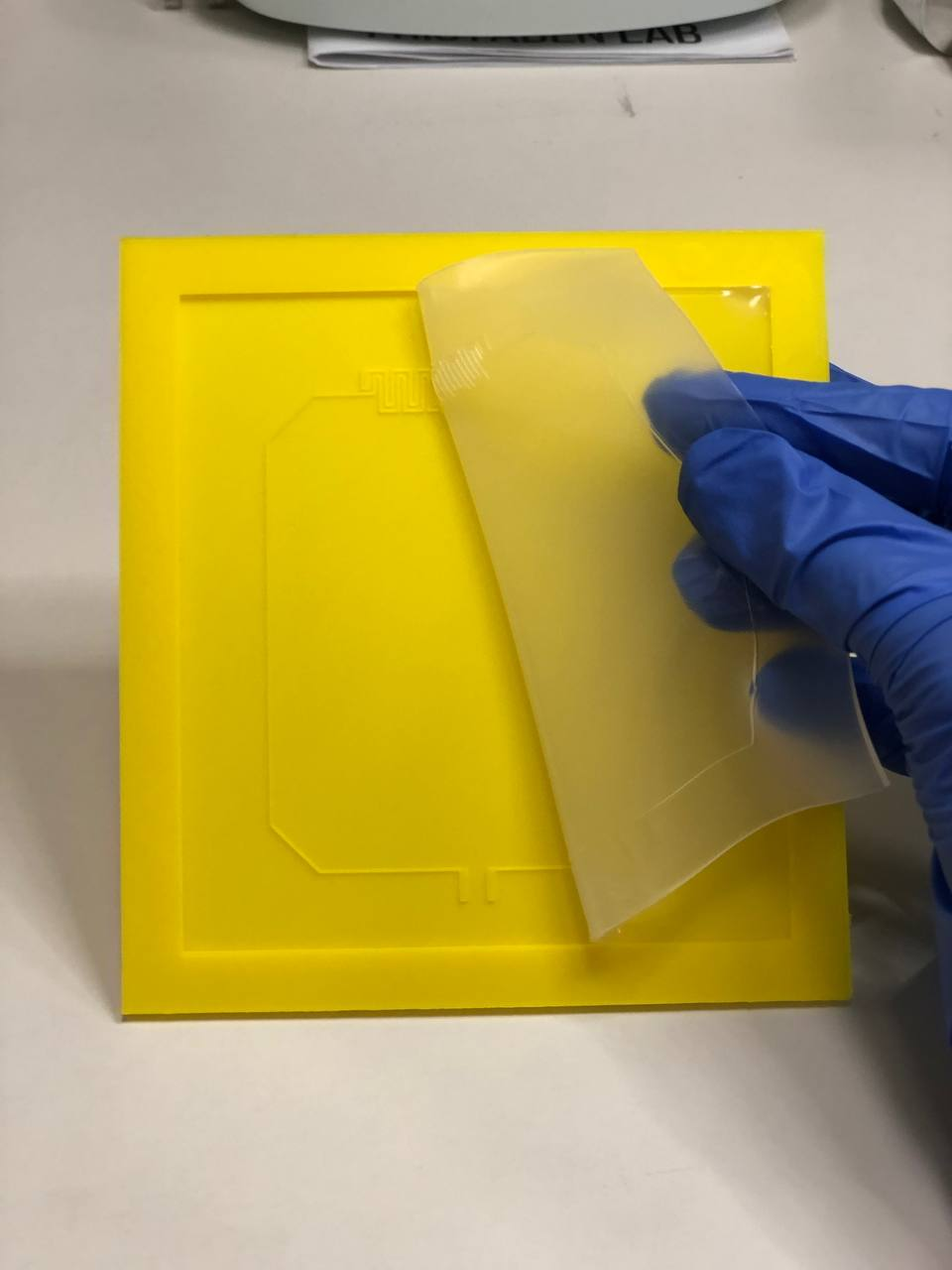

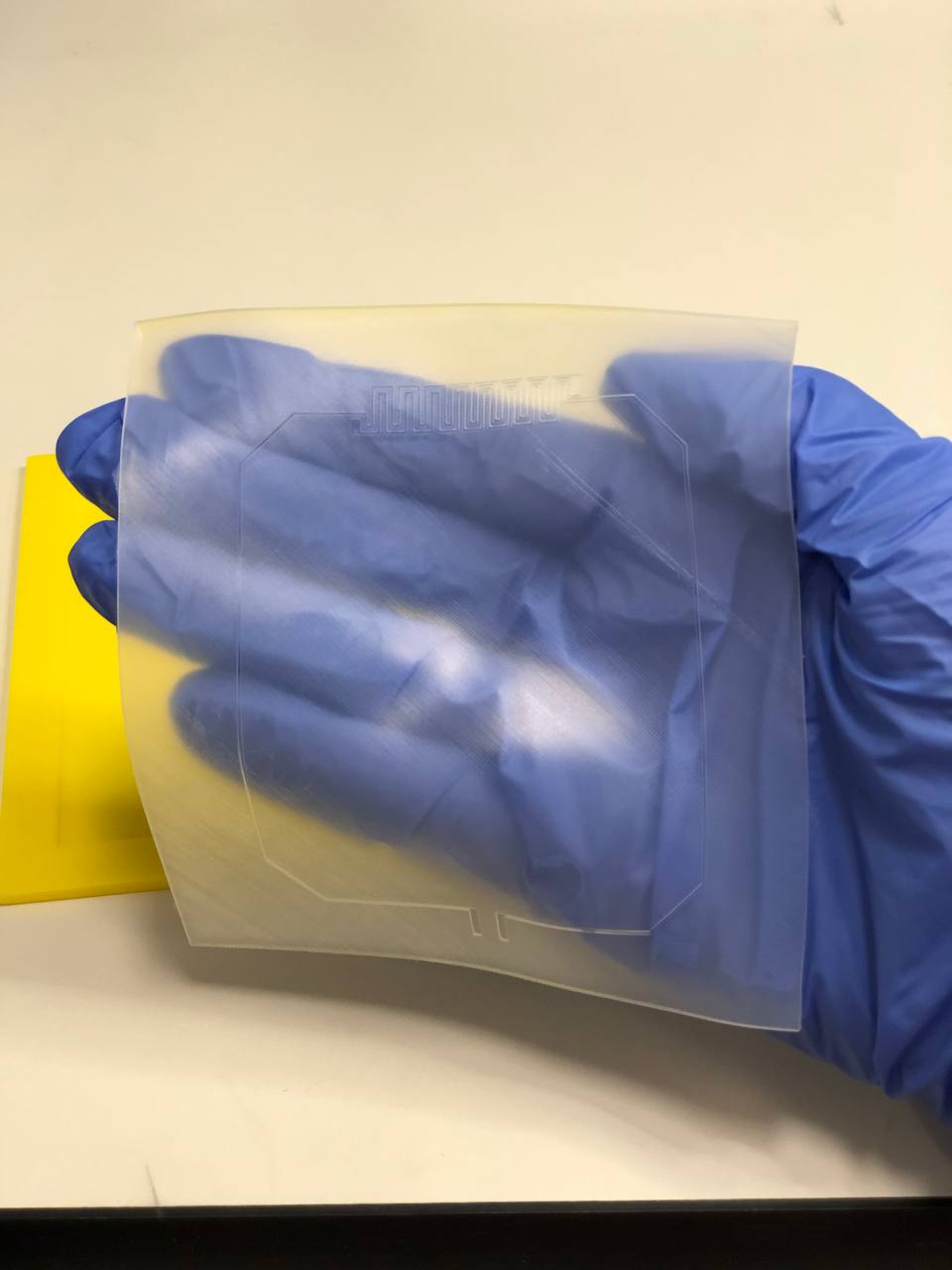

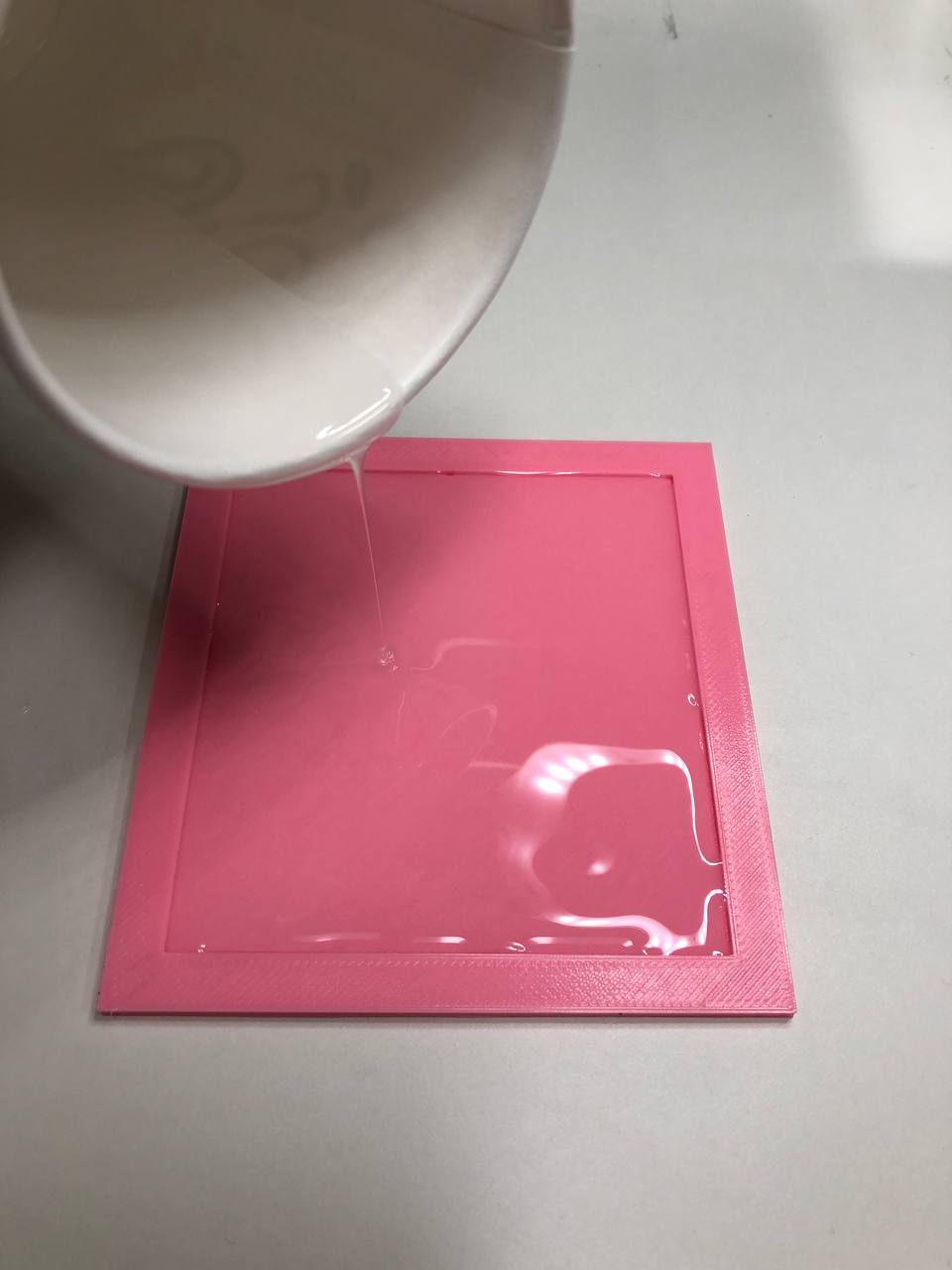

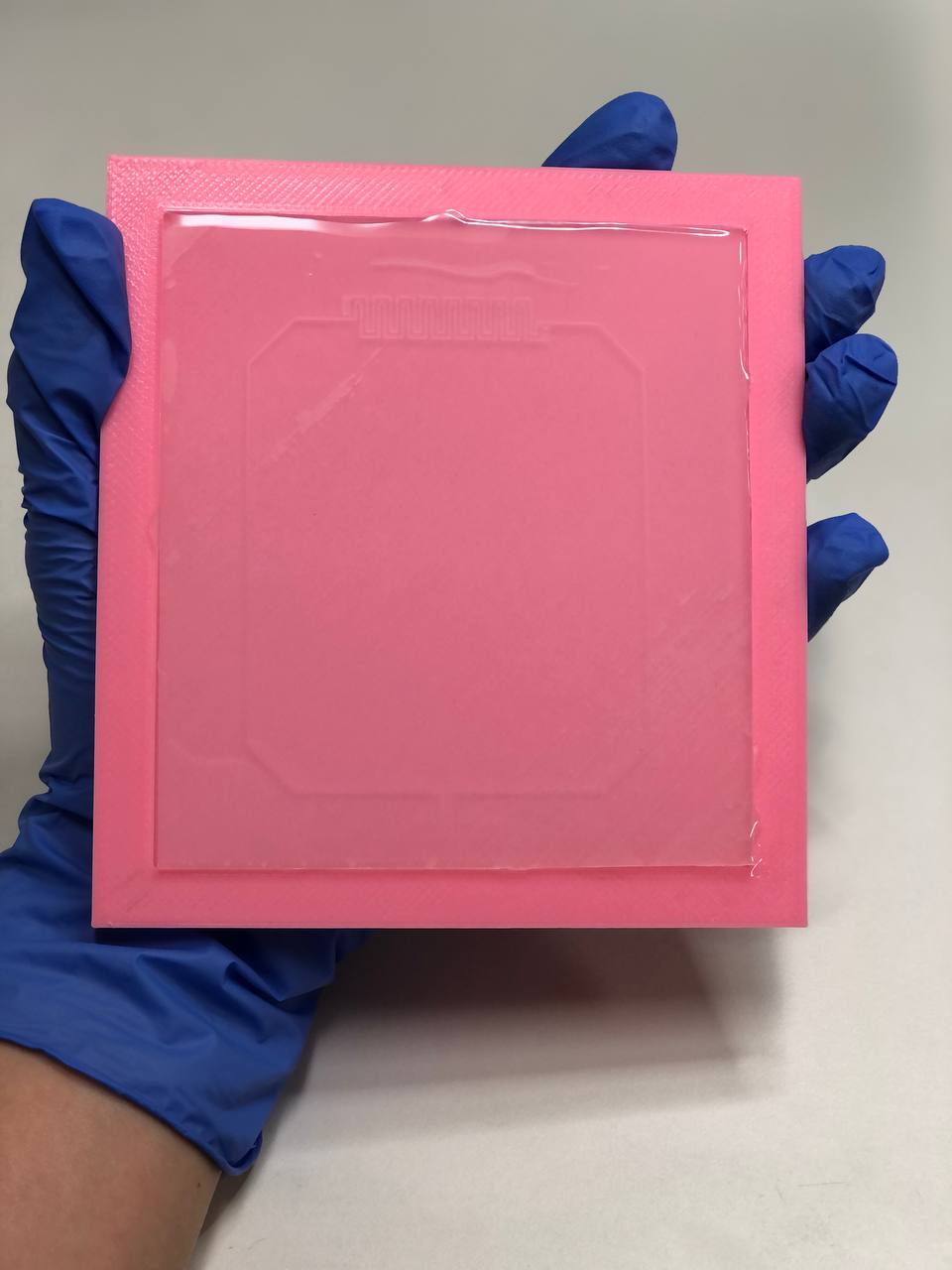

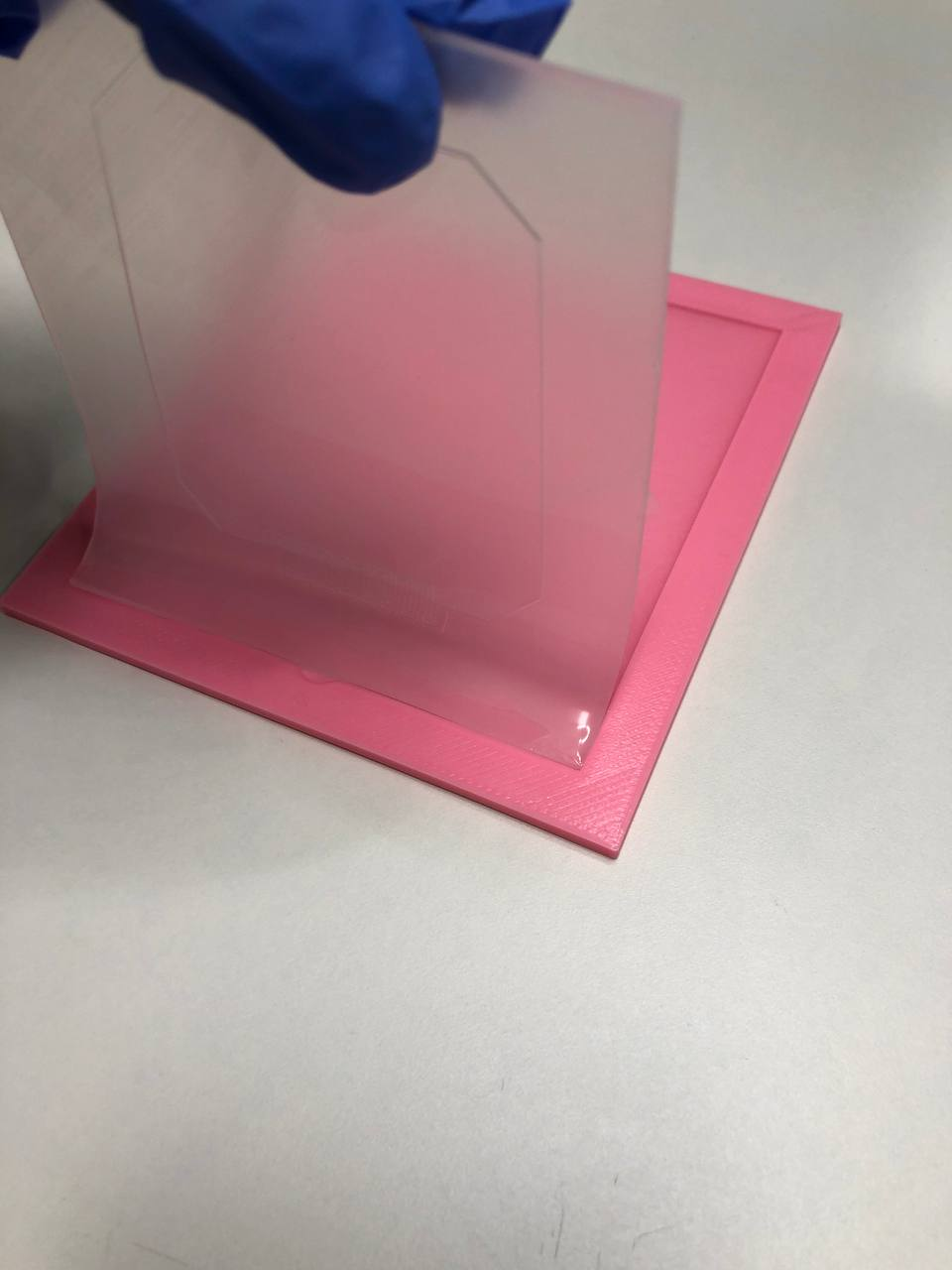

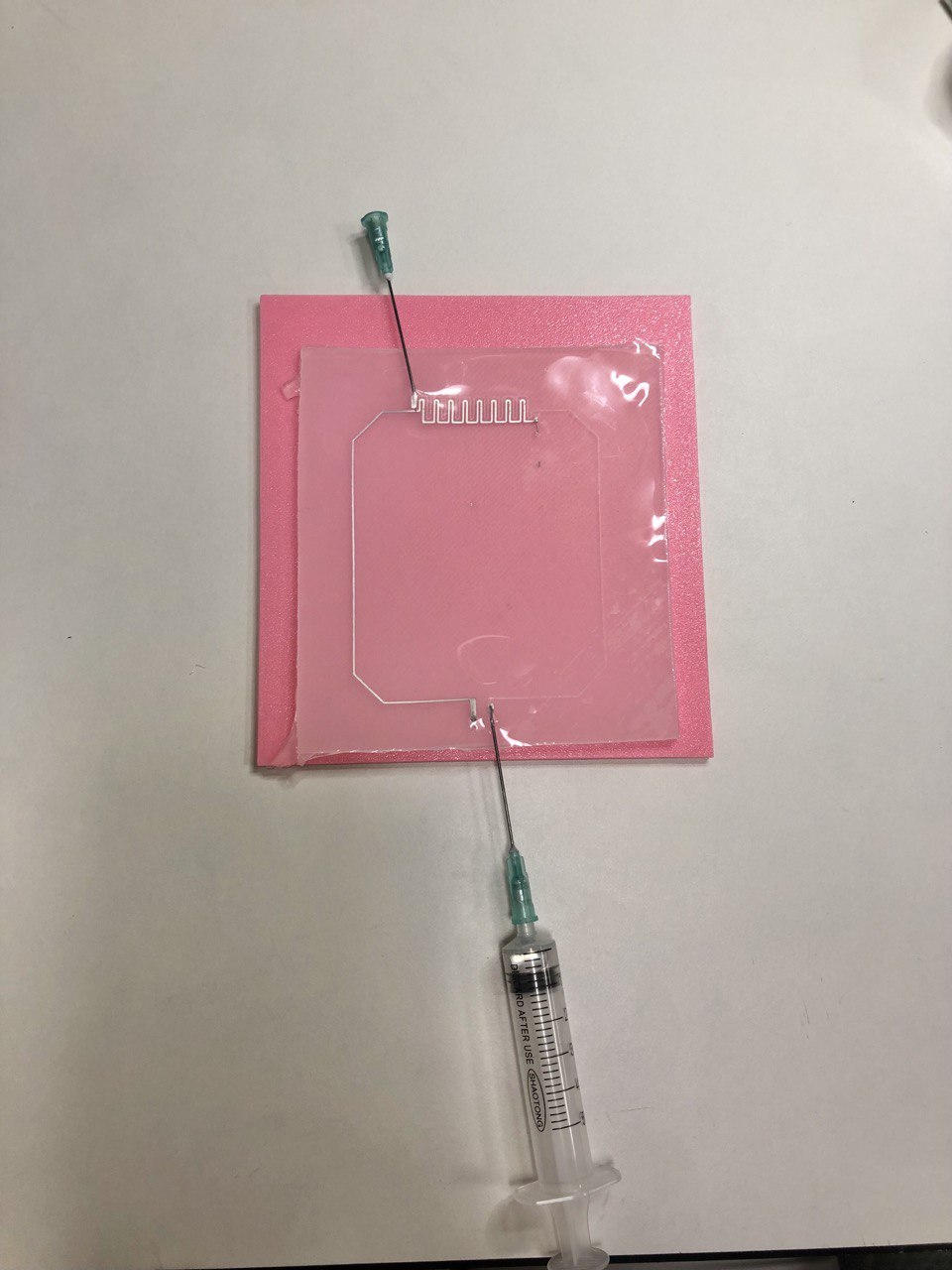

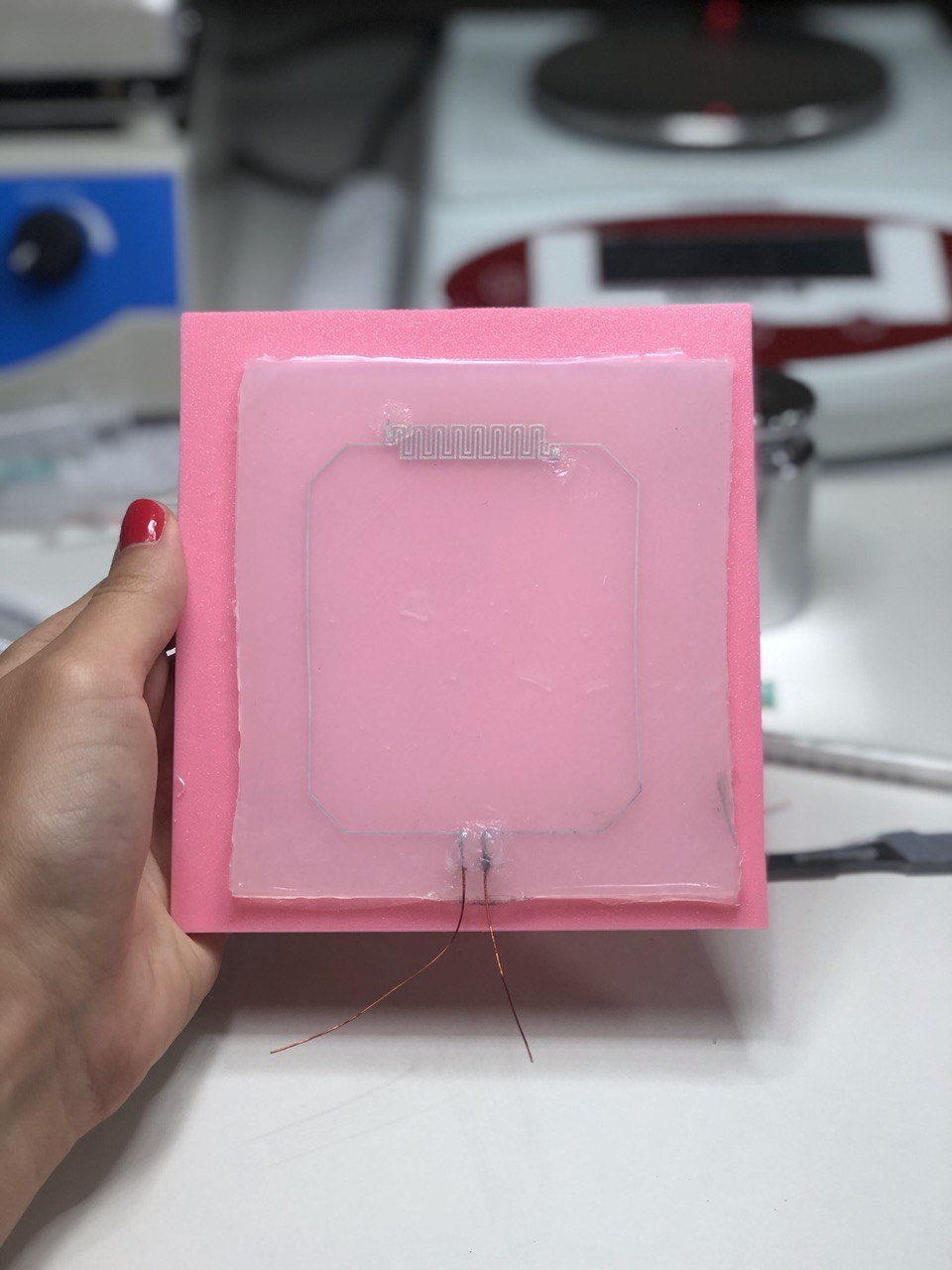


(a)

(b)

(c)

(d)

(e)

(f)

(g)

(h)

(i)

(j)

(k)

(l)

Figure S2. A step-by-step coil fabrication process.

Figure S3 shows a circuit schematic used as feeding circuitry for the proposed coil. It consists of two tuning capacitors and a lattice balun. The capacitor values are as follows: $C_{t}=24pF$, $C_{b}=27pF$, $L_{b}=56nH$. The custom preamplifier has a noise figure of <0.5dB with a gain of 28dB at 127.7MHz and input impedance of >1kΩ (GE Healthcare).


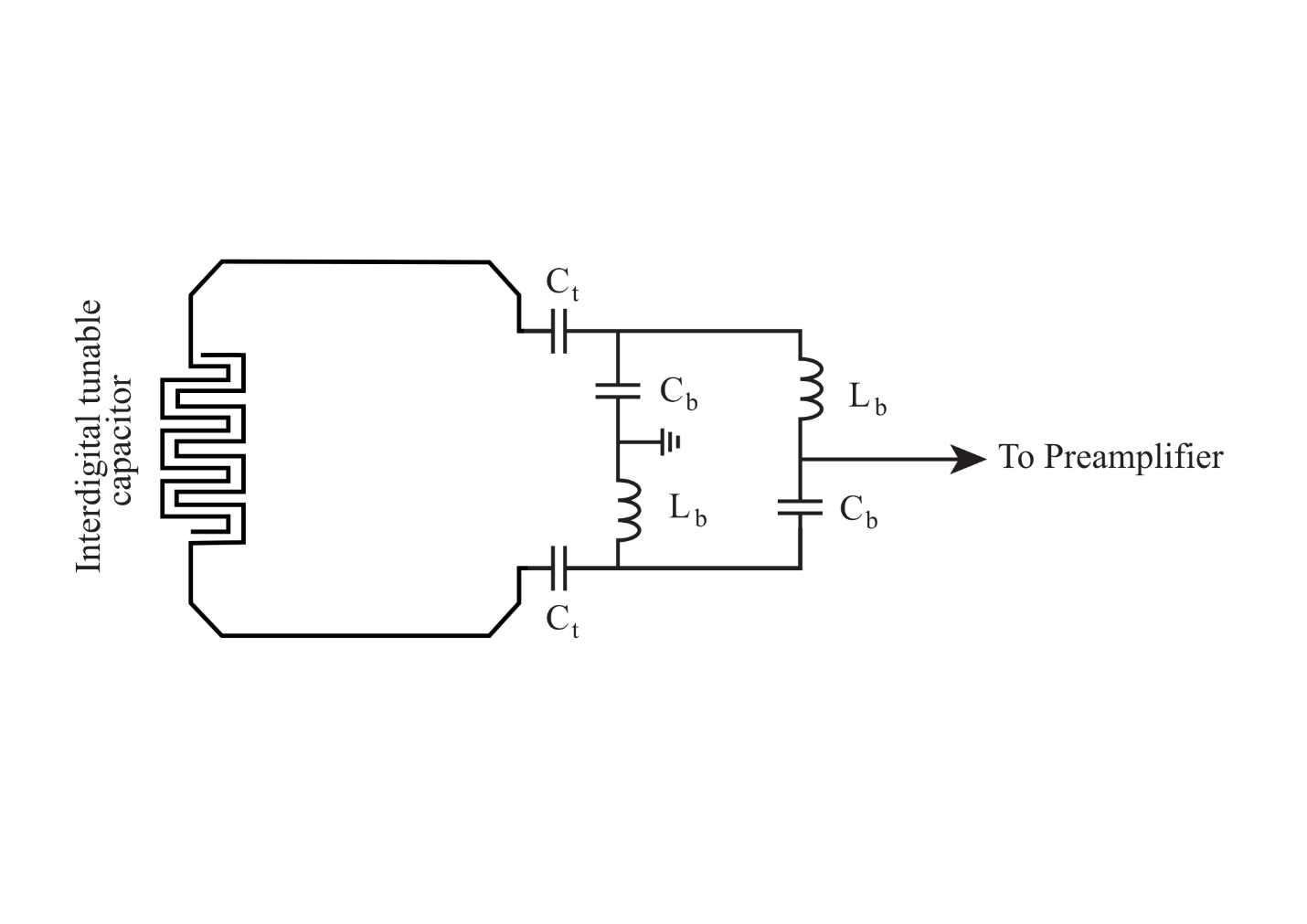


Figure S3. Schematic of the proposed RF receive coil.

Supporting videos:

V1. Stretchability demonstration of the proposed coil.

V2. In vivo images (SNR maps) produced with the proposed coil when stretching from 0% to 50%.

References:

[1] Sigma-Aldrich, Gallium-Indium eutectic; Available from: <https://www.sigmaaldrich.com/US/en/product/aldrich/495425>.

[2] Ecoflex by Smooth-On; Available from: <https://www.smooth-on.com/product-line/ecoflex/>.

[3] Eddings MA, Johnson MA, Gale BK. Determining the optimal PDMS–PDMS bonding technique for microfluidic devices. Journal of Micromechanics and Microengineering 2008;18(6):067001.

[4] BACOENG Chamber and Pump Kit; 2021. Available from: <https://bacoeng.com/products/vacuum-chamber-kit?variant=32055709827>.
